# Supplementary material for: Five-Year Trends in Potential Drug Interactions with Direct-Acting Oral Anticoagulants in Patients with Atrial Fibrillation: An Australian-Wide Study
Source: J Clin Med. 2020 Nov 5;9(11):3568. doi: 10.3390/jcm9113568 (PMC7694473; doi:10.3390/jcm9113568)
Supplement: Supplementary file 1 [file jcm-09-03568-s001.pdf]

Table S1. Trends in potential drug interactions with DOACs in patients with atrial fibrillation.

Year	Medication Class	Total	With potential drug interaction	
		n	*n*	% (95% CI)
2014	All	5334	2884	45.9 (44.6–47.3)
	SSRIs/SNRIs	5334	772	14.5 (13.5–15.4)
	NSAIDs	5334	617	11.6 (10.7–12.4)
	CCB	5334	602	11.3 (10.4–12.1)
	Amiodarone	5334	367	6.9 (6.2–7.6)
2015	All	8281	3663	44.2 (43.2–45.3)
	SSRIs/SNRIs	8281	1184	14.3 (13.5–15.1)
	NSAIDs	8281	879	10.6 (10.0–11.3)
	CCB	8281	903	10.9 (10.2–11.6)
	Amiodarone	8281	485	5.9 (5.4–6.4)
2016	All	11,575	4847	41.9 (41.0–42.8)
	SSRIs/SNRIs	11,575	1682	14.5 (13.9–15.2)
	NSAIDs	11,575	1198	10.4 (9.8–10.9)
	CCB	11,575	1143	9.9 (9.3–10.4)
	Amiodarone	11,575	775	6.7 (6.3–7.2)
2017	All	15,414	6242	40.5 (39.7–41.3)
	SSRIs/SNRIs	15,414	2260	14.7 (14.1–15.2)
	NSAIDs	15,414	1465	9.5 (9.0–10.0)
	CCB	15,414	1440	9.3 (8.9–9.8)
	Amiodarone	15,414	963	6.3 (5.9–6.6)
2018	All	19,196	7653	39.9 (39.2–40.6)
	SSRIs/SNRIs	19,196	2849	14.8 (14.3–15.3)
	NSAIDs	19,196	1864	9.7 (9.3–10.1)
	CCB	19,196	1687	8.8 (8.4–9.2)
	Amiodarone	19,196	1250	6.5 (6.2–6.9)

All interacting medications with direct-acting oral anticoagulants (DOACs) included non-steroidal anti-inflammatory drugs (NSAIDs), antiplatelet agents, macrolide antibiotics, oral azole antifungals, HIV protease inhibitors, calcium channel blockers (CCBs), antiarrhythmic agents (amiodarone), antiepileptics (phenytoin, carbamazepine, valproate, levetiracetam, primidone) or selective serotonin/norepinephrine reuptake inhibitors (SSRIs/SNRIs).
